# Prediction of the Effects of Liraglutide on Kidney and Cardiovascular Outcomes Based on Short-Term Changes in Multiple Risk Markers

**DOI:** 10.3389/fphar.2022.786767

**Published:** 2022-04-13

**Authors:** Sok Cin Tye, Sieta T. de Vries, Johannes F. E. Mann, Meir Schechter, Ofri Mosenzon, Petra Denig, Hiddo J. L. Heerspink

**Affiliations:** ^1^ Department of Clinical Pharmacy and Pharmacology, University Medical Center Groningen, Groningen, Netherlands; ^2^ KfH Kidney Center, Munich, Germany; ^3^ Department of Medicine, Friedrich Alexander University, Erlangen, Germany; ^4^ Faculty of Medicine, Hebrew University of Jerusalem, Jerusalem, Israel; ^5^ Diabetes Unit, Hadassah Hebrew University Hospital, Jerusalem, Israel

**Keywords:** liraglutide, diabetes, risk markers, kidney outcomes, cardiovascular outcomes

## Abstract

**Aims:** The LEADER trial demonstrated that the glucagon-like peptide-1 receptor agonist (GLP1-RA) liraglutide reduces kidney and cardiovascular (CV) risk in patients with type 2 diabetes. We previously developed a Parameter Response Efficacy (PRE) score that translates multiple short-term risk marker changes, from baseline to first available follow-up measurement, into a predicted long-term drug effect on clinical outcomes. The objective of this study was to assess the accuracy of the PRE score in predicting the efficacy of liraglutide in reducing the risk of kidney and CV outcomes.

**Methods:** Short-term changes in glycated hemoglobin (HbA1c), systolic blood pressure (BP), urinary-albumin-creatinine-ratio (UACR), hemoglobin, body weight, high-density-lipoprotein (HDL) cholesterol, low-density-lipoprotein (LDL) cholesterol, and potassium were monitored in the LEADER trial. Associations between risk markers and kidney or CV outcomes were established using a multivariable Cox proportional hazards model in a separate pooled database of 6,355 patients with type 2 diabetes. The regression coefficients were then applied to the short-term risk markers in the LEADER trial to predict the effects of liraglutide on kidney (defined as a composite of doubling of serum creatinine or end-stage kidney disease) and CV (defined as a composite of non-fatal myocardial infarction, non-fatal stroke, and CV death) outcomes.

**Results:** Liraglutide compared to placebo reduced HbA1c (1.4%), systolic BP (3.0 mmHg), UACR (13.2%), body weight (2.3 kg), hemoglobin (2.6 g/L), and increased HDL-cholesterol (0.01 mmol/L) (all *p*-values <0.01). Integrating multiple risk marker changes in the PRE score resulted in a predicted relative risk reduction (RRR) of 16.2% (95% CI 13.7–18.6) on kidney outcomes which was close to the observed RRR of 15.5% (95% CI -9.0–34.6). For the CV outcome, the PRE score predicted a 7.6% (95% CI 6.8–8.3) RRR, which was less than the observed 13.2% (95% CI 3.2–22.2) RRR.

**Conclusion:** Integrating multiple short-term risk markers using the PRE score adequately predicted the effect of liraglutide on the composite kidney outcome. However, the PRE score underestimated the effect of liraglutide for the composite CV outcome, suggesting that the risk markers included in the PRE score do not fully capture the CV benefit of liraglutide.

## Introduction

In the last years, glucagon-like peptide 1 receptor agonists (GLP1-RA) including liraglutide, semaglutide, albiglutide, dulaglutide, and efpeglenatide have been shown to decrease the risk of cardiovascular (CV) events in patients with type 2 diabetes and atherosclerotic CV disease or at high CV risk (Gerstein et al., 2021; Tuttle et al., 2021). In addition, GLP1-RA have been shown to slow the progression of kidney function decline particularly in patients with chronic kidney disease (Tuttle et al., 2018; Kristensen et al., 2019; van Ruiten et al., 2021).

Emerging studies show that the improvement in glycemic control only mediates a small part of the kidney protective effect of GLP1-RA. In the LEADER and the SUSTAIN-6 trial, the reduction in HbA1c with liraglutide and semaglutide only explained 25% of the kidney protective effect of these therapies (Mann et al., 2021). GLP1-RA exert multiple effects on risk markers of CV and kidney disease progression such as effects on blood pressure, body weight, and albuminuria. These effects may contribute to the long-term protective effect of these therapies (Muskiet et al., 2017; Nauck et al., 2021). In addition, GLP1-RA may also exert protective effects by promoting natriuresis and diuresis, down-regulate pro-inflammatory, and oxidative stress pathways, and exert direct beneficial effects on endothelial function (Drucker, 2018; Sposito et al., 2018; Kawanami and Takashi, 2020).

Recognising the multiple effects of GLP1-RA in mediating kidney and CV protection, we hypothesized that a multivariable risk score, which integrates the short-term effects of drugs, would outperform single surrogate in the prediction of drug effects on long-term clinical outcomes. The multiple Parameter Response Efficacy (PRE) score was previously developed to predict the long-term effect of angiotensin receptor blockers, endothelin receptor antagonists, and sodium glucose co-transporter 2 (SGLT2) inhibitors on kidney and CV outcomes (Smink et al., 2014a; Smink et al., 2014b; Schievink et al., 2015; Schievink et al., 2016; Idzerda et al., 2020). In the accompanying article, we describe the utility of the PRE score to predict the efficacy of the SGLT2 inhibitor empagliflozin in patients with established CV disease (Tye et al., 2021). The objective of this study is to assess the accuracy of the PRE score in estimating the effect of GLP1-RA liraglutide on kidney and CV outcomes in patients at high CV risk. Second, we applied the PRE score to predict the effect of the GLP1-RA semaglutide in the ongoing FLOW trial, a long-term kidney outcome trial to assess the long-term efficacy and safety of semaglutide which is expected to report in 2024.

## Materials and Methods

### Data Sources and Population

The PRE score was used to estimate the effect of liraglutide on kidney and CV outcomes. The PRE score was intended as a flexible algorithm which can be used to any drug or population by fitting the regression coefficients from the multivariable Cox proportional hazards model onto the short-term risk markers measured in an independent population. In this study, the short-term risk markers and outcome relationships were established at baseline in a background dataset consisting of a pooled population of patients with type 2 diabetes at high risk of kidney events or with an established CV disease from the ALTITUDE, RENAAL, and IDNT trials (Supplementary Table 1). The pooled database consisted of 6,355 patients with type 2 diabetes at high risk of kidney events or with an established CV disease in whom a total of 1,129 (17.8%) composite kidney outcomes, and 794 (12.5%) primary composite CV outcomes were recorded during follow-up. The designs and primary outcomes for these trials have been previously published (Ruggenenti et al., 1997; Lewis et al., 2001; Parving et al., 2012). The estimated beta-coefficients were then applied to all patients in the LEADER trial to assess the liraglutide-induced relative risk reduction (RRR) using the PRE score.

In addition, we selected patients from the LEADER trial who fulfilled the inclusion criteria of the ongoing FLOW trial which is designed to assess the effect of semaglutide on major kidney and CV death outcomes in patients with diabetic kidney disease (NCT03819153). In this subgroup from the LEADER trial, we estimated the effect of liraglutide on the composite kidney and CV death outcomes to predict future results of the FLOW trial.

### Risk Markers Selection

Variables that were measured in the intention-to-treat population in the LEADER trial and previously identified as risk markers for kidney or CV outcomes were used, i.e., glycated hemoglobin (HbA1c), systolic blood pressure (BP), urinary-albumin-creatinine ratio (UACR), body weight, hemoglobin (Hb), high-density-lipoprotein (HDL) cholesterol, low-density-lipoprotein (LDL) cholesterol, and serum potassium (K).

### Outcome Definition

Since the PRE score was initially developed to predict drug efficacy on kidney outcomes, we first predicted the effect of liraglutide on the kidney outcome which was a secondary outcome in the LEADER trial and defined as a composite of confirmed doubling of serum creatinine (DSCR) or end stage kidney disease (ESKD). The CV outcome in the LEADER trial was defined as a composite of non-fatal myocardial infarction, non-fatal stroke, or CV death. For prediction of the effect of GLP1-RA in the ongoing FLOW trial, we used the primary outcome of the trial defined as a composite of a sustained decline in estimated glomerular filtration rate (eGFR) by 50%, ESKD, or CV death.

### Statistical Analysis

A multivariable Cox proportional hazards model was used to estimate the beta-coefficients associated with kidney or CV outcomes in the background dataset. These beta-coefficients were then applied to the baseline and 6-months (or first available) risk marker measurement for patients in the LEADER trial, to estimate the risk of specific outcomes at both time points in the placebo or liraglutide arm, h(t) = h_o_(t) $\;e\sum^{\beta 1*X1}$ where event rate at time t is a product of baseline hazard (h_o_(t)) and the sum of the linear function of the estimated *β* coefficients and the respective risk marker measurement(s) (*X*). The mean difference in the predicted risk in the liraglutide arm, adjusted for the mean difference in their predicted risk at the placebo arm, represents the PRE score and reflects an estimated kidney or CV risk reduction induced by liraglutide treatment. To estimate the 95% confidence intervals (CI) on the predicted RRR, 100 sets of coefficients were generated from independent normal distributions based on the estimated regression coefficients and their standard error from the Cox proportional hazards model.

In the LEADER trial dataset, several risk markers were not available at 6-months, therefore we used those available at subsequent visits (12-months: UACR, HDL-cholesterol, LDL-cholesterol; 24-months: hemoglobin and K). To assess the impact of the selection of risk markers at different time-points, we performed an additional analysis using all biomarkers at 24-months. To further assess the robustness of our findings we also performed a complete case analysis based on the risk marker changes between baseline and first follow-up measurement (Supplementary Table 2).

For the prediction of the FLOW trial outcomes, we performed additional simulation analyses with a simulated range of liraglutide induced responses on albuminuria, HbA1c, systolic BP, and body weight since the effect of semaglutide on the various risk markers may differ from the effects of liraglutide in the LEADER trial. We simulated these markers because they are important risk markers of kidney and CV outcomes and showed the most significant reductions following liraglutide treatment. Simulations were performed by shifting the distribution of the responses in these risk markers. This was done by selecting different proportions of patients with a response in the risk marker where response was defined as a reduction more or equal than the median.

Based on the missing at random assumption, we imputed all variables which contain missing data using Multiple Imputation by Chained-Equation (using the R package “MICE”, version 3.11.0) where predictive mean matching, a semi-parametric approach that replaces missing values based on multivariable regression was used (White et al., 2011). Covariate distributions were checked visually to ensure reasonably imputed values. For variables which are normally distributed, means and standard deviations are reported. For UACR, medians with first and third interquartile ranges are reported due to the non-normal distribution and natural log-transformation was applied for this variable in the Cox proportional hazards model. Categorical variables are described in frequencies and percentages. A two-sided *p*-value smaller than 0.05 was considered statistically significant. All statistical analyses were performed in R version 4.1.1 (R Project for Statistical Computing, http://www.r-project.org).

## Results

In the LEADER trial, a total of 9,340 patients were randomly assigned to receive liraglutide (N = 4,672) or placebo (N = 4,668) and were included in the intention-to-treat population. The participants of the LEADER trial were characterized by high CV risk in general. At baseline, there were 7,598 (81.3%) participants who had an established CV disease. The mean HbA1c was 8.7%, systolic BP was 135.9 mmHg, eGFR was 79.1 ml/min/1.73 m^2^ and 1,982 (21.2%) patients had an eGFR <60 ml/min/1.73 m^2^. The median UACR at baseline was 24.8 mg/g. Demographic and clinical characteristics of the patients were well-balanced between the treatment and placebo group (Table 1).

**TABLE 1:** Baseline characteristics of patients included in the background dataset and in the LEADER trial.

Characteristic	Background population (N = 6,355)	Total population in the LEADER trial (N = 9,340)	
		Placebo (N = 4,672)	Treatment (N = 4,668)
Age (years)	61.0 (9.0)	68.4 (7.2)	64.2 (7.2)
Female, n (%)	2,128 (33.5)	1,680 (36.0)	1,657 (35.5)
Race, n (%)			
Caucasian	3,529 (55.5)	3,622 (77.5)	3,616 (77.5)
Black	569 (9.0)	407 (8.7)	370 (7.9)
Asian	1,527 (24.0)	465 (10.0)	471 (10.1)
Others	730 (11.5)	178 (3.8)	211 (4.5)
eGFR (ml/min/1.73m^2^)	51.2 (22.5)	79.3 (21.8)	78.9 (22.4)
Glycated hemoglobin (%)	8.1 (1.7)	8.7 (1.5)	8.7 (1.6)
Systolic BP (mmHg)	144.8 (20.0)	135.9 (17.7)	135.9 (17.8)
UACR (mg/g)	276.9 [54.5, 1193.9]	26.2 [7.2, 156.6]	23.3 [7.0, 134.4]
Weight (kg)	83.4 (19.8)	91.6 (20.8)	91.9 (21.2)
Hemoglobin (g/L)	128 (18.7)	137.1 (14.8)	137.1 (15.3)
HDL-cholesterol (mmol/L)	1.2 (0.4)	1.2 (0.3)	1.2 (0.3)
LDL-cholesterol (mmol/L)	3.1 (1.3)	2.3 (0.9)	2.3 (0.9)
Potassium (mmol/L)	4.6 (0.5)	4.5 (0.5)	4.5 (0.5)

For numerical variables which are normally distributed, data is presented as mean (SD). For UACR with a skewed distribution, median [IQR] is presented. Categorical variables are presented as frequency (%). BP, blood pressure; UACR, urinary-albumin-creatinine-ratio; HDL, high-density-lipoprotein; LDL, low-density-lipoprotein. Estimated glomerular filtration rate (eGFR) was calculated using the Chronic Kidney Disease Epidemiology Collaboration (CKD-EPI) formula which is in accordance to the LEADER trial protocol (Levey et al., 2009; Marso et al., 2016a).

### Short-Term Changes in Risk Markers

**FIGURE 1:**
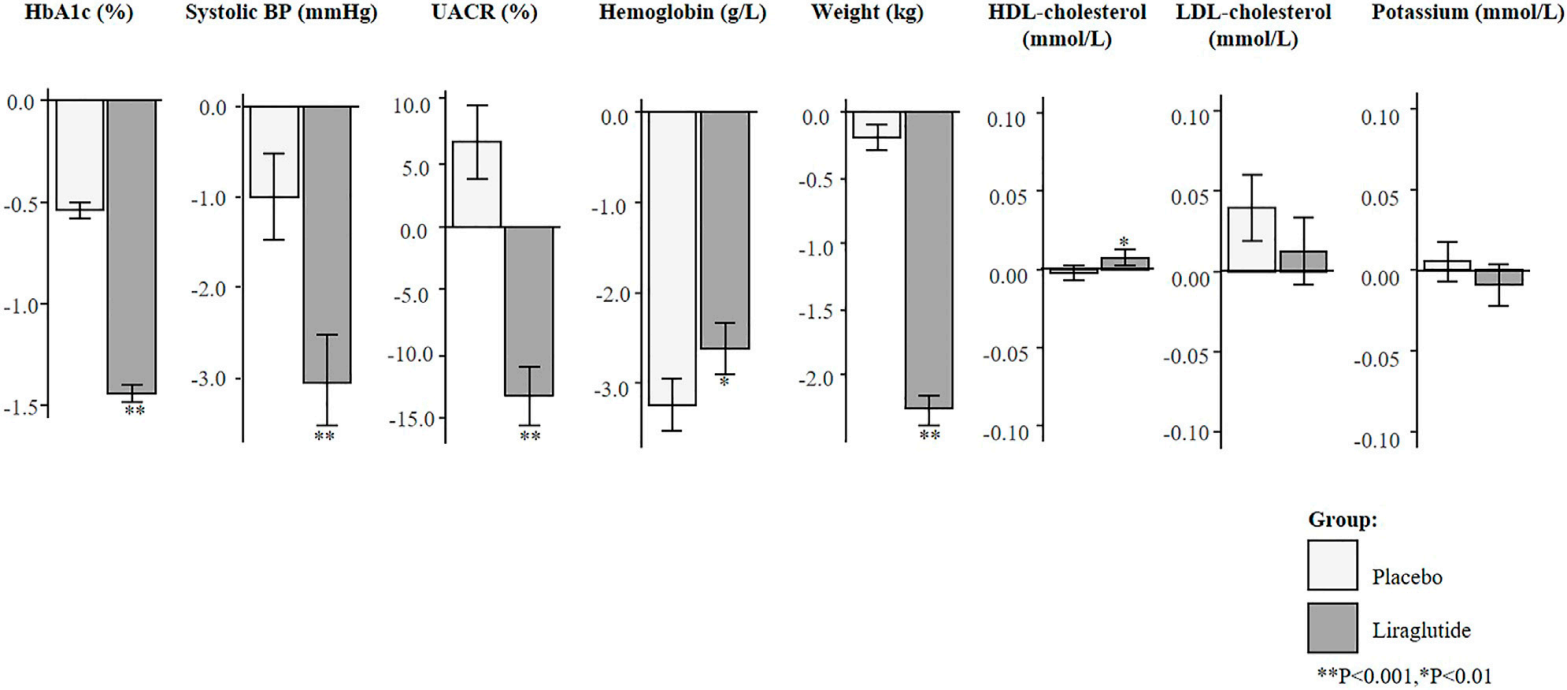
Mean changes in risk markers from baseline to 6-months (or first follow-up) measurement in the imputed total population of the LEADER trial (N = 9,340). Changes are presented as mean with 95% confidence intervals, for the placebo and liraglutide group. HbA1c, glycated hemoglobin; BP, blood pressure; UACR, urinary-albumin-creatinine-ratio; HDL, high-density-lipoprotein; LDL, low-density-lipoprotein.

Short-term risk marker changes at 6-months (or first available) measurement observed in the LEADER trial are given in Figure 1. Treatment with liraglutide significantly reduced HbA1c (1.4%), systolic BP (3.0 mmHg), UACR (13.2%), and body weight (2.3 kg) (*p* < 0.001). A decrease in hemoglobin (2.6 g/L) and mild elevation of HDL-cholesterol (0.01 mmol/L) were observed (*p* < 0.01). The direction and magnitude of the effect of liraglutide on individual risk marker change was similar in the complete case analysis and the additional analysis using biomarker changes from baseline to 24-months (Supplementary Figures 1,2).

### Observed and PRE Score Predicted Treatment Effect

**FIGURE 2:**
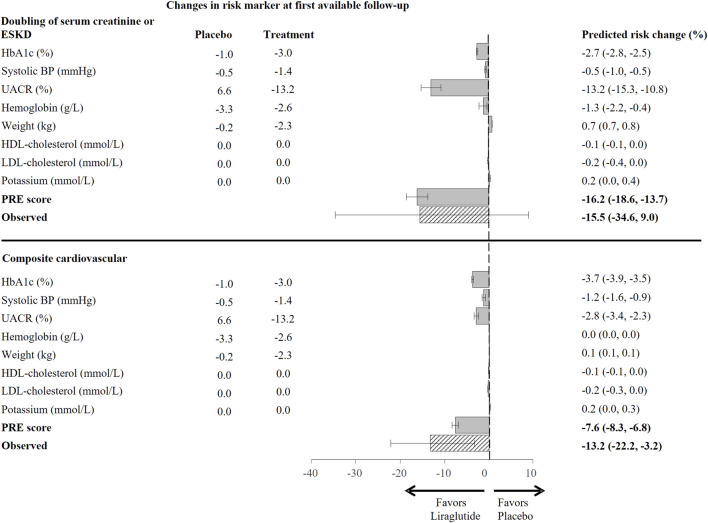
Predicted risk change for the composite kidney (doubling of serum creatinine or ESKD) and the composite cardiovascular (non-fatal myocardial infarction, non-fatal stroke, or cardiovascular death) outcome in the total population based on single risk marker as well as the PRE score, using risk markers changes from baseline to 6-months or first available follow up (N = 9,340). Bars indicate estimates of the mean change in relative risk for specific outcomes with 95% confidence intervals, as compared to placebo. HbA1c, glycated hemoglobin; BP, blood pressure; UACR, urinary-albumin-creatinine-ratio; HDL, high-density-lipoprotein; LDL, low-density-lipoprotein; PRE score, Parameter Response Efficacy score; ESKD, end stage kidney disease.

During 3.8 years of follow-up in the LEADER trial (N = 9,340), there were 237 (2.5%) patients who developed a kidney outcome, and 1,302 (13.9%) who developed a CV outcome. The observed RRR of liraglutide was 15.5% (95% CI −9.0–34.6) for the kidney outcome, and 13.2% (95% CI 3.2–22.2%) for the CV outcome (Figure 2). The prediction of the treatment effect of liraglutide based on single surrogates caused an underestimation of the overall treatment effect on kidney or CV outcomes. For instance, based on the observed placebo corrected HbA1c reduction, it was estimated that liraglutide would reduce the risk of the kidney outcome by 2.7% (95% CI 2.5–2.8) and the CV outcome by 3.7% (95% CI 3.5–3.9). Based on albuminuria reduction alone, the predicted RRR of liraglutide on the kidney outcome was 13.2% (95% CI 10.8–15.5) and 2.8% (95% CI 2.3–3.4) for the CV outcome. Integrating the multiple short-term risk marker changes using the PRE score resulted in a predicted RRR of 16.2% (95% CI 13.7–18.6) for the kidney outcome and RRR of 7.6 (95% CI 6.8–8.3) for the CV outcome.

In the complete case analysis, the predicted RRR for the kidney outcome was similar to the observed, a RRR 17.1% (95% CI 14.2–19.9) *vs.* 15.8% (95% CI -11.6–36.5), respectively. The PRE score underestimated the RRR for the CV outcome [predicted 7.7% (95% CI 6.9–8.6) *vs.* observed 13.9% (95% CI 2.4–24.0)] (Supplementary Figure 3).

In the analysis using risk marker changes from baseline to 24-months (N = 9,340), the observed RRR for the kidney outcome was 15.5% (95% CI −9.0–34.6), while the PRE score predicted a RRR of 14.7% (95%CI 11.9–17.4) (Supplementary Figure 4). For the CV outcome, the PRE score underestimated the effect of liraglutide, the observed RRR was 13.2% (3.2–22.2) while the PRE score predicted a RRR of 5.6% (95% CI 4.7–6.5).

### Prediction of the FLOW Trial Outcomes

**FIGURE 3:**
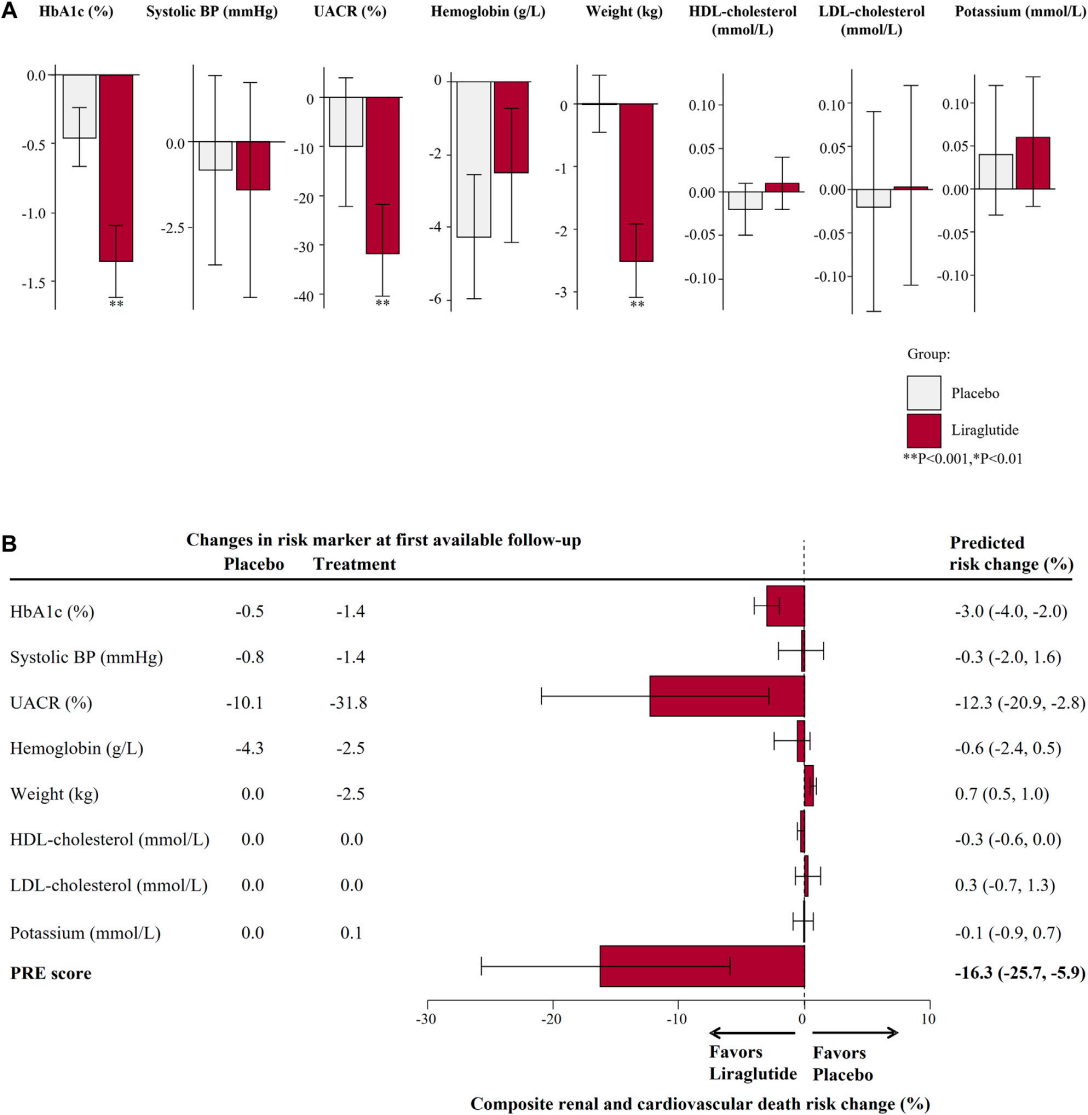
**(A)** Mean changes in risk markers from baseline to 6-months (or first follow-up) measurement in the subset of the LEADER Trial according to the FLOW trial inclusion criteria (N = 327). Changes are presented as mean with 95% confidence intervals, for the placebo and liraglutide group. **(B)** Predicted risk change for the primary composite kidney or cardiovascular death outcomes for the ongoing FLOW trial based on single risk marker changes as well as the PRE score. HbA1c, glycated hemoglobin; BP, blood pressure; UACR, urinary-albumin-creatinine-ratio; HDL, high-density-lipoprotein; LDL, low-density-lipoprotein; PRE score, Parameter Response Efficacy score.

There were 327 (3.5%) patients from the LEADER trial who met the inclusion criteria of the ongoing FLOW trial. In this subgroup, the mean age was 66.7 years, mean eGFR was 38.9 ml/min/1.73 m^2^ and median UACR was 1,086.9 mg/g (Supplementary Table 3). The baseline characteristics of these individuals were well matched between the placebo and liraglutide group. Liraglutide reduced HbA1c (1.4%), UACR (31.8%), and body weight (2.5 kg), *p* < 0.001 (Figure 3A).

**FIGURE 4:**
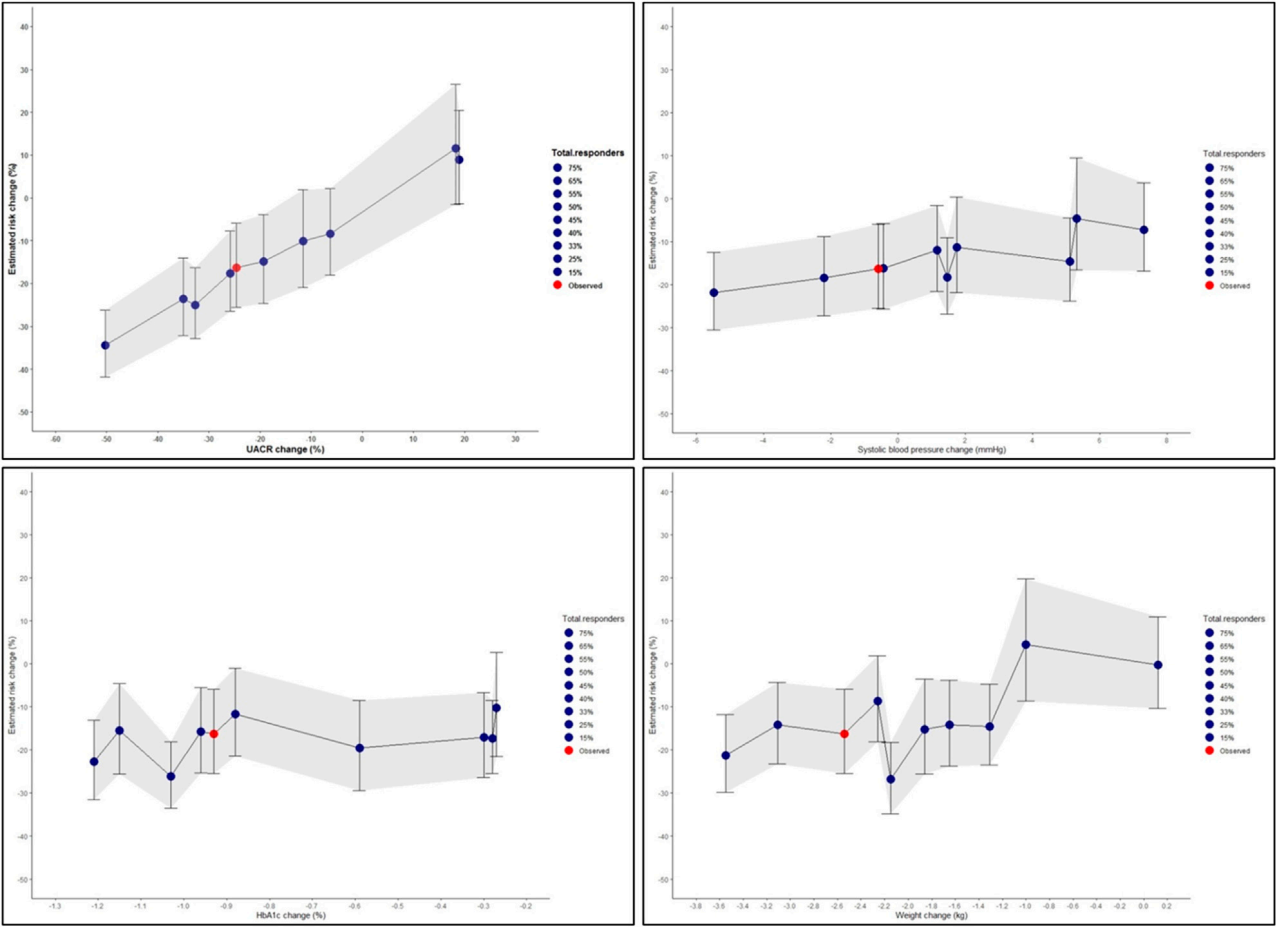
Simulated risk marker changes and the effect on the composite kidney or cardiovascular death outcomes for the ongoing FLOW trial based on the selected LEADER trial population. The shaded area indicates the 95% confidence intervals for the simulated risk marker changes. Risk prediction was estimated from short-term risk marker changes in the FLOW population (N = 327) chosen from the LEADER trial, and simulated values for UACR, systolic blood pressure, HbA1c, and body weight. The red dot indicates the PRE score predicted risk change in the FLOW trial without enrichment with responders. The blue dots represent changes observed in the different proportion of responders and non-responders for each riskmarker. HbA1c, glycated hemoglobin; UACR, urinary-albumin-to-creatinine ratio.

Using the PRE score, we estimated that semaglutide as compared to placebo in the FLOW trial will lead to a RRR of 16.3% (95% CI 5.9–25.7) for the composite kidney or CV death outcome (Figure 3B). Our simulations demonstrated that the effect of liraglutide is predominantly driven by changes in UACR whereby with larger or smaller UACR changes, the effect of liraglutide on the kidney or CV death outcome were markedly higher or lesser. To achieve a RRR in the primary outcome of the FLOW trial by 20%, we estimated that at least a 31% decrease in UACR is required (Figure 4).

## Discussion

In this study, we demonstrated that integrating short-term changes in multiple risk markers resulted in a predicted RRR of 16.2% for the kidney outcome which was of similar magnitude to the RRR observed in the LEADER trial. These results suggest that integrating short-term effects of liraglutide on multiple cardiorenal risk markers adequately predicts its long-term efficacy on the kidney outcome. The PRE score underestimated the CV protective effect of liraglutide suggesting that mechanistic pathways beyond those captured by the risk markers included in the PRE score may explain the CV protective effect of liraglutide.

GLP1-RA are originally developed as glucose-lowering agents for the treatment of type 2 diabetes. GLP1-RA are approved by regulatory agencies based on their HbA1c lowering efficacy (Marso et al., 2016a; Mann et al., 2017). However, our analyses and other studies have demonstrated that changes in HbA1c do not completely account for the long-term protective effects. In the AWARD-7 trial for example, dulaglutide compared to insulin treatment resulted in similar effects on HbA1c but reduced the rate of eGFR decline and the risk of ESKD in patients with diabetic kidney disease (Tuttle et al., 2018). Furthermore, a pooled analysis of exenatide studies demonstrated that exenatide reduced albuminuria compared to other glucose lowering agents at equal glycemic control suggesting that potential renal benefits are in part independent of glycemic effects (van der Aart-van der Beek et al., 2020).

Which other mechanisms could explain the long-term protective effects of GLP1-RA? The mechanisms underlying the long-term protective effects of incretin-based therapies are not completely understood but several possibilities exist. It is well known that GLP1-RA decrease multiple CV risk markers. These beneficial effects may explain long-term CV protective effects. However, a mediation analysis from the LEADER trial demonstrated that the CV protective effect of liraglutide was only partly mediated by traditional risk markers suggesting, as in the current analyses, that mechanistic pathways not captured by routinely used clinical chemistry markers may be involved (Buse et al., 2020). Indeed, mechanistic studies have shown that GLP1-RA enhance sodium excretion by inhibition of the sodium-hydrogen exchange 3 transporter (Skov et al., 2013; Muskiet et al., 2016; Tonneijck et al., 2019). Increased sodium excretion may lead to improved CV and kidney outcomes through a variety of mechanistic pathways. Second, GLP1-RA improve endothelial function which may be an underlying pathway of CV and renal protection (Koska et al., 2010; Koska et al., 2015). Moreover, reductions in inflammation and oxidative stress have been observed with GLP-1RA in preclinical and clinical studies thereby potentially reducing vascular tissue injury (Zhou et al., 2014; Li et al., 2017; Helmstädter et al., 2020). Further study is needed to determine whether incorporating biomarkers representing these pathways improve the accuracy of the PRE-score to predict CV protective GLP-1RA effects.

In our study, albuminuria reduction represented a strong predictor of the long-term CV and kidney effect of liraglutide. Earlier studies with angiotensin receptor blockers, endothelin receptor antagonists, and sodium glucose co-transporter 2 inhibitors have shown that albuminuria reduction is strongly associated with subsequent reductions in major kidney outcomes. (Wang et al., 2018; Heerspink et al., 2019; McGuire et al., 2021). There is strong and consistent evidence that GLP1-RA reduce albuminuria. In the AWARD-7 trial, dulaglutide treatment significantly reduced albuminuria in a dose-related manner, with a larger effect observed among patients with elevated baseline UACR (>300 mg/g). (Tuttle et al., 2018). In a post-hoc analysis of the ELIXA trial, lixisenatide reduced albuminuria with more pronounced effects in patients with micro- or macroalbuminuria (Muskiet et al., 2018). Efpeglenatide also reduced albuminuria by 21% in patients with type 2 diabetes with a history of CV disease or current kidney disease (Gerstein et al., 2021). Since early treatment effects on albuminuria are associated with long-term drug effects on clinical outcomes, these data support dedicated kidney outcome trials to assess the long-term efficacy and safety of GLP1-RA.

The FLOW trial is the first dedicated trial to assess the effect of a long-acting GLP1-RA for kidney or CV death protection among type 2 diabetes patients with varying degree of kidney impairment and albuminuria status (Novo Nordisk A/S, 2021). We estimated using the observed biomarker changes that treatment with GLP1-RA may lead to a primary endpoint reduction in the FLOW trial by 16.3%. We acknowledge that only 327 patients in the LEADER trial fulfilled the inclusion criteria for the FLOW trial and therefore these estimations should be interpreted with caution. In addition, we note that these predictions are dependent upon the actual patients enrolled in the FLOW trial and their actual risk marker changes. Based on previous studies we expect that semaglutide reduces UACR by 25–34% (for doses between 0.5 and 1 mg/week) among type 2 diabetes patients (Marso et al., 2016b; Mann et al., 2020). In our simulation analysis, assuming a 30% reduction in UACR compared to the 13.2% reduction we observed in LEADER, we estimate that semaglutide would reduce the risk of the primary composite outcome of the FLOW trial by at least 20%. The final results of the FLOW trial will provide a clearer answer whether the estimation using the PRE score is accurate.

There are several limitations in our study. First, the effect of liraglutide on the kidney and CV outcomes showed a wide confidence interval which limited our ability to make direct comparisons of the PRE score predicted effect with the observed treatment effect. Additional kidney and CV outcome studies with GLP1-RA, such as the FLOW and SOUL (NCT03914326) trials with semaglutide, are therefore required to more definitively confirm the validity of the PRE score. Second, to keep the PRE score relatively easy to apply, it focused on modifiable risk factors and does not account for changes in non-modifiable risk factors (e.g., demographics), changes in medication use, or complex interactions between risk factors. Third, not all parameters were available in the 6-months and thus we have used the baseline and first available follow-up measurement to account for the drug-induced risk marker changes, with additional analyses conducted with the complete cases and risk markers measured at baseline and 24-months. Fourth, the primary endpoint for the FLOW trial is based on a relatively small subgroup as we note that the majority of the participants in the LEADER trial had only mild-to moderate chronic kidney disease. The analysis is thus exploratory and post-hoc in nature and therefore can only be considered hypothesis generating. Finally, the background population consisted of patients with different characteristics than those enrolled in the LEADER trial. However, we have previously shown that this does not impact the predictive performance of the PRE score (Idzerda et al., 2021).

In conclusion, integrating multiple short-term GLP1-RA induced drug effects in an algorithm (PRE score) led to a prediction of the effect of liraglutide on major kidney outcomes which was similar as the observed drug effect in the LEADER trial. However, the PRE score underestimated the effect of liraglutide on CV outcomes. Additional research is warranted to determine if adding novel biomarkers to the PRE score would improve prediction of the CV protective effects of GLP1-RA.

## Data Availability

Request to access datasets and syntaxes for this study can be directed to the corresponding author.
