# Supplementary material for: Prediction of the Effects of Liraglutide on Kidney and Cardiovascular Outcomes Based on Short-Term Changes in Multiple Risk Markers
Source: Front Pharmacol. 2022 Apr 13;13:786767. doi: 10.3389/fphar.2022.786767 (PMC9044907; doi:10.3389/fphar.2022.786767)

Supplementary Material

**Supplementary** **Table 1.** Description of trials included as background dataset and the LEADER trial dataset

| Trial name and ClinicalTrials.gov identifier | Trial description | Median follow-up time (years) | Intervention | No. of patients included in imputed case analysis/ Total patients enrolled in each trial | No. of patients included in complete case analysis/ Total patients enrolled in each trial |
| --- | --- | --- | --- | --- | --- |
| *Trials included as background dataset* | | | | |  |
| RENAAL [NCT00308347] | RCT in patients with non-insulin dependent diabetes, hypertension, and urinary protein>1+ on dipstick | 3.4 | Losartan (50 to 100 mg once daily) or placebo | 1136/1513 | 1471/1513 |
| IDNT trial [NCT00317915] | RCT in patients with diabetes, hypertension, and nephropathy | 2.6 | Irbesartan (300 mg daily), amlodipine (10 mg daily), or placebo. | 1471/1715 | 1703/1715 |
| ALTITUDE  [NCT00549757] | RCT in patients with diabetes, with evidence of albuminuria and history of cardiovascular disease | 2.7 | Aliskiren 300 mg/day, or placebo as adjunct to ACEi/ARB | 3181/8561 | 3181/8561 |
| *The LEADER trial dataset* | | | | |  |
| LEADER  [NCT01179048] | RCT in patients with diabetes aged 50 and above with concomitant cardiovascular diseases, or patients with diabetes aged 60 and above with at least one cardiovascular risk factor | 3.8 | Liraglutide 1.8mg  versus placebo | 9340/9340 | 6905/9340 |

RENAAL, Reduction of Endpoints in NIDDM with the Angiotensin II Antagonist Losartan; IDNT, Irbesartan Diabetic Nephropathy Trial; ALTITUDE, Aliskiren Trial in Type 2 Diabetes Using Cardiorenal Endpoints; LEADER, Liraglutide Effect and Action in Diabetes: Evaluation of Cardiovascular Outcome Results; RCT, randomized controlled trial; ACEi, angiotensin converting enzyme inhibitor; ARB, angiotensin II receptor blocker.

**Supplementary** **Table 2.** Baseline characteristics of patients included in the background dataset and complete cases in the LEADER trial dataset

| **Characteristic** | **Background population**  **(n=6355)** | **Complete cases without missing for HbA1c, SBP, UACR, body weight, hemoglobin, HDL-cholesterol, LDL-cholesterol, and potassium at baseline and first available follow-up measurement (N=6905)** | | |
| --- | --- | --- | --- | --- |
|  |  | **Overall**  **(N=6905)** | **Placebo**  **(N=3460)** | **Treatment**  **(N=3445)** |
| Age (years) | 61.0 (9.0) | 64.5 (7.2) | 64.7 (7.2) | 64.4 (7.2) |
| Female, n (%) | 2128 (33.5) | 2320 (33.6) | 1164 (33.6) | 1156 (33.6) |
| Race, n (%) |  |  |  |  |
| Caucasian | 3529 (55.5) | 5255 (7601) | 2622 (75.8) | 2633 (76.4) |
| Black | 569 (9.0) | 618 (9.0) | 330 (9.5) | 288 (8.4) |
| Asian | 1527 (24.0) | 749 (10.8) | 372 (10.8) | 377 (10.9) |
| Others | 730 (11.5) | 283 (4.1) | 136 (3.9) | 147 (4.3) |
| eGFR (ml/min/1.73m2) * | 51.2 (22.5) | 78.5 (22.5) | 78.6 (22.3) | 78.4 (22.7) |
| Glycated hemoglobin (%) | 8.1 (1.7) | 8.7 (1.5) | 8.7 (1.5) | 8.8 (1.6) |
| Systolic BP (mmHg) | 144.8 (20.0) | 136.6 (17.9) | 136.5 (17.8) | 136.7 (18.0) |
| UACR (mg/g) | 276.9 [54.5, 1193.9] | 23.3 [8.4, 89.4] | 24.3 [8.5, 94.2] | 22.4 [8.3, 86.7] |
| Weight (kg) | 83.4 (19.8) | 91.5 (21.1) | 91.5 (20.9) | 91.5 (21.3) |
| Hemoglobin (g/L) | 128 (18.7) | 136.8 (15.3) | 136.8 (15.0) | 136.8 (15.5) |
| HDL-cholesterol (mmol/L) | 1.2 (0.4) | 1.2 (0.3) | 1.2 (0.3) | 1.2 (0.3) |
| LDL-cholesterol (mmol/L) | 3.1 (1.3) | 2.3 (0.90 | 2.3 (0.9) | 2.3 (0.9) |
| Potassium (mmol/L) | 4.6 (0.5) | 4.5 (0.5) | 4.5 (0.5) | 4.5 (0.5) |

For numerical variables which are normally distributed, data is presented as mean (SD). For UACR with a skewed distribution, median [IQR] is presented. Categorical variables are presented as frequency (%).UACR, urinary-albumin-creatinine-ratio; BP, blood pressure; HDL, high-density-lipoprotein; LDL, low-density-lipoprotein. *The estimated glomerular filtration rate (eGFR) was calculated using the Chronic Kidney Disease Epidemiology Collaboration (CKD-EPI) formula in accordance to the LEADER trial protocol.

**Supplementary Table 3.** Baseline characteristics of patients from the LEADER trial with eGFR ≥50 ml/min/1.73m^2^ and ≤75 ml/min/1.73m^2^ (CKD-EPI equation) and UACR >300 mg/g and <5000 mg/g or ≥50 and <50 ml/min/1.73m^2^ and UACR >100 mg/g and <5000 mg/g, whom fulfilled the inclusion criteria of the FLOW trial

| **Characteristic** | **Patients with eGFR ≥50 ml/min/1.73m^2^ and ≤75ml/min/1.73m^2^ (by the CKD-EPI equation) and UACR >300 mg/g and <5000 mg/g or ≥50 and <50 ml/min/1.73m^2^ and UACR >100 mg/g and <5000 mg/g from the LEADER trial (N=327)** | | |
| --- | --- | --- | --- |
|  | **Overall**  **(N=327)** | **Placebo**  **(N=169)** | **Treatment**  **(N=158)** |
| Age (years) | 66.7 (7.7) | 66.9 (7.9) | 66.6 (7.5) |
| Female, n (%) | 131 (40.1) | 70 (41.4%) | 61(38.6) |
| Race, n (%) |  |  |  |
| Caucasian | 231 (70.6) | 116 (68.6) | 115 (72.7) |
| Black | 38 (11.6) | 23 (13.6) | 15 (9.5) |
| Asian | 35 (10.7) | 17 (10.1) | 18 (11.4) |
| Others | 23 (7.0) | 13 (7.7) | 10 (6.3) |
| eGFR (ml/min/1.73m2)* | 38.9 (7.0) | 38.6 (7.3) | 39.3 (6.7) |
| Glycated hemoglobin (%) | 8.8 (1.7) | 8.8 (1.7) | 8.7 (1.6) |
| Systolic BP (mmHg) | 141.7 (22.0) | 141.7 (21.7) | 141.7 (22.4) |
| UACR (mg/g) | 1086.9 [583.7, 1927.6] | 1108.5 [599.8, 1947.6] | 1047.6 [572.0, 1914.1] |
| Weight (kg) | 91.1 (21.6) | 91.6 (22.1) | 90.6 (21.2) |
| Hemoglobin (g/L) | 127.3 (16.7) | 127.0(17.5) | 127.6 (15.8) |
| HDL-cholesterol (mmol/L) | 1.2 (0.3) | 1.2 (0.4) | 1.2 (0.3) |
| LDL-cholesterol (mmol/L) | 2.5 (1.0) | 2.5 (1.0) | 2.4 (1.0) |
| Potassium (mmol/L) | 4.6 (0.6) | 4.6 (0.6) | 4.6 (0.6) |

For numerical variables which are normally distributed, data is presented as mean (SD). For UACR with a skewed distribution, median [IQR] is presented. Categorical variables are presented as frequency (%). UACR, urinary-albumin-creatinine-ratio; BP, blood pressure; HDL, high-density-lipoprotein; LDL, low-density-lipoprotein. *The estimated glomerular filtration rate (eGFR) was calculated using the Chronic Kidney Disease Epidemiology Collaboration (CKD-EPI) formula in accordance to the LEADER trial protocol.

**Supplementary** **Figure 1.** Mean changes in risk markers from baseline to 6-month/ first follow up measurement in the LEADER trial population for complete case analyses (N=6905). Changes are presented as mean with 95% confidence interval, for the placebo and liraglutide group. HbA1c, glycated hemoglobin; UACR, urinary-albumin-creatinine-ratio; BP, blood pressure; HDL, high-density-lipoprotein; LDL, low-density-lipoprotein.


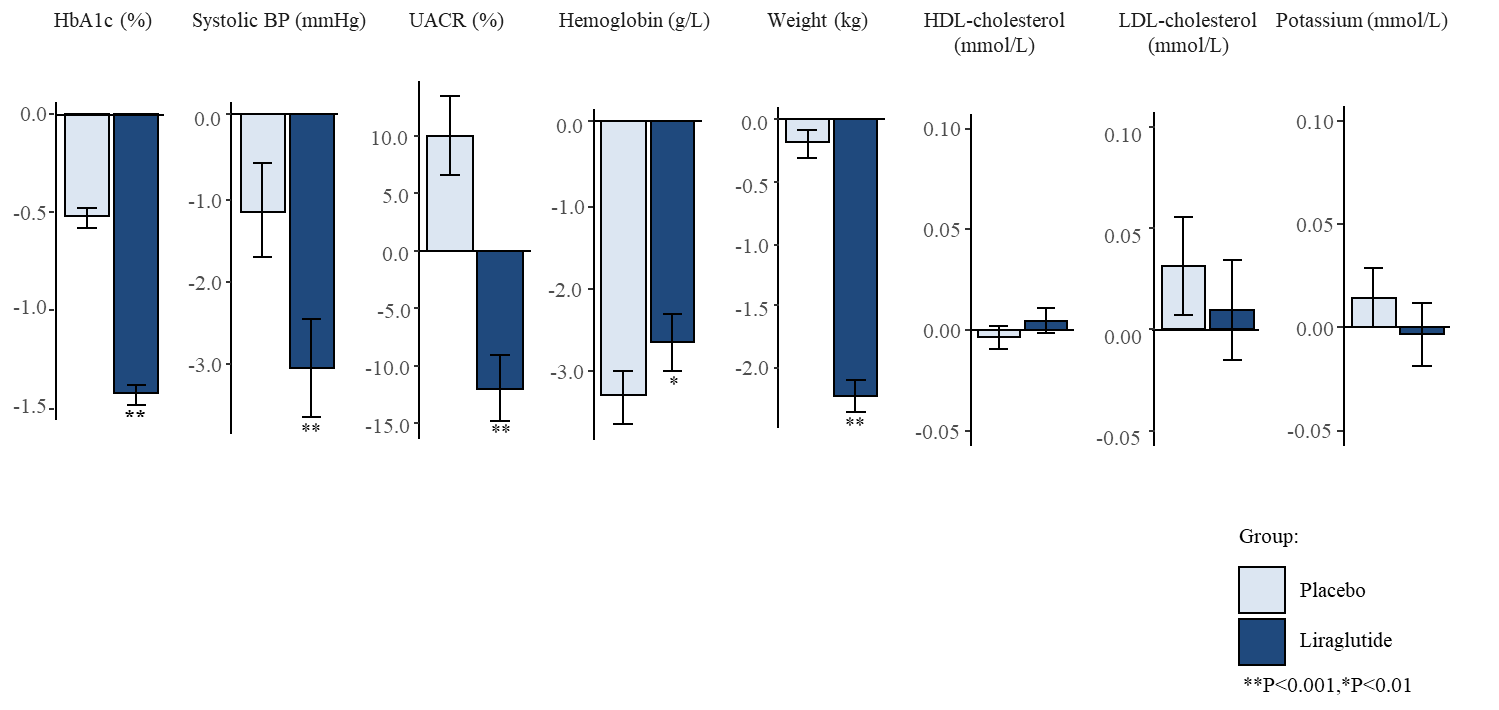


**Supplementary Figure 2.** Mean changes in risk markers from baseline to 24-month measurement in the LEADER trial total population (N=9340). Changes are presented as mean with 95% confidence interval, for the placebo and liraglutide group. HbA1c, glycated hemoglobin; UACR, urinary-albumin-creatinine-ratio; BP, blood pressure; HDL, high-density-lipoprotein; LDL, low-density-lipoprotein.


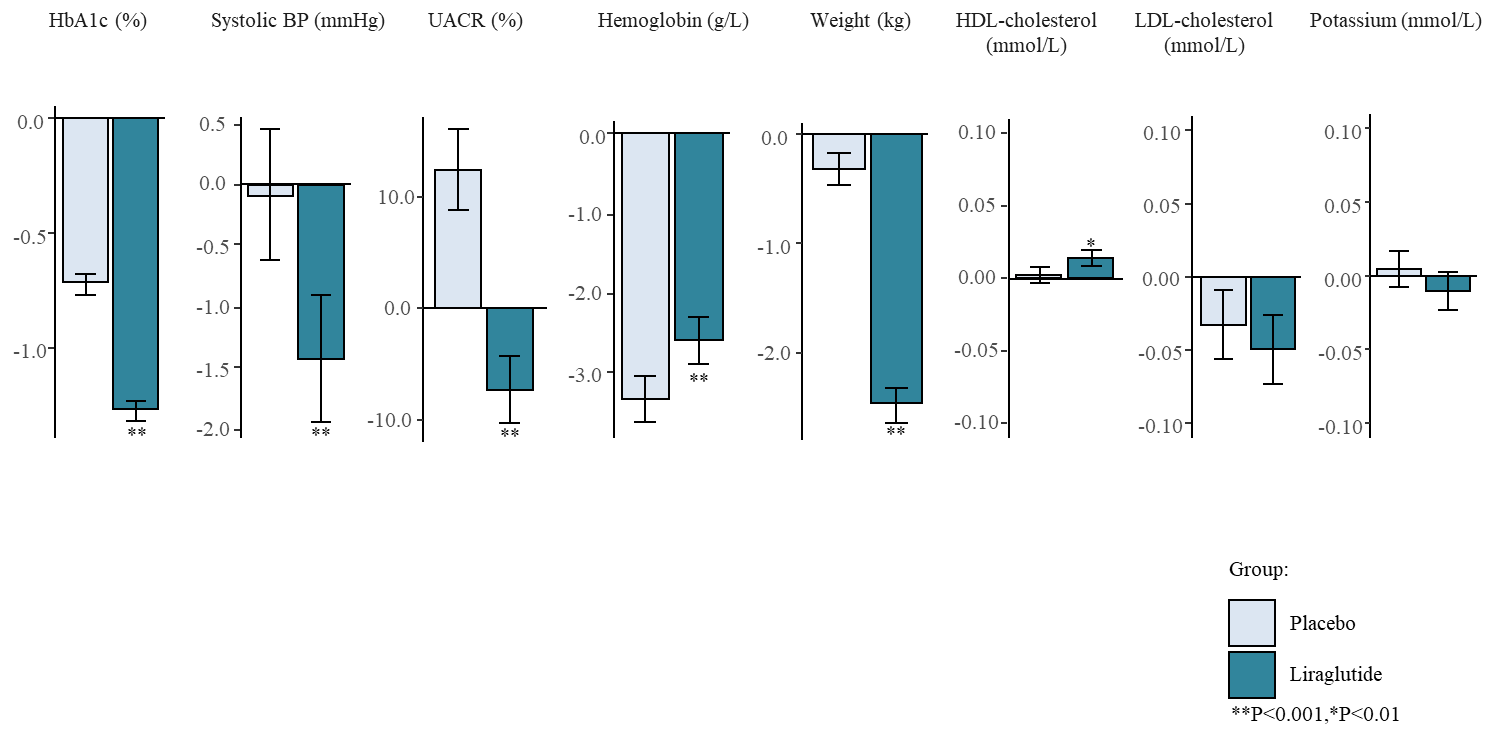


**Supplementary Figure 3.** Predicted risk change for the composite kidney (top) and the composite cardiovascular (bottom) outcomes in the complete case analyses (N=6905) with all risk markers measured at baseline and 6-month/ first available follow-up. Bars indicate estimates of the mean change in relative risk for specific outcomes with 95% confidence interval, as compared to placebo. HbA1c, glycated hemoglobin; UACR, urinary-albumin-creatinine-ratio; BP, blood pressure; HDL, high-density-lipoprotein; LDL, low-density-lipoprotein; PRE score, Parameter Response Efficacy score.


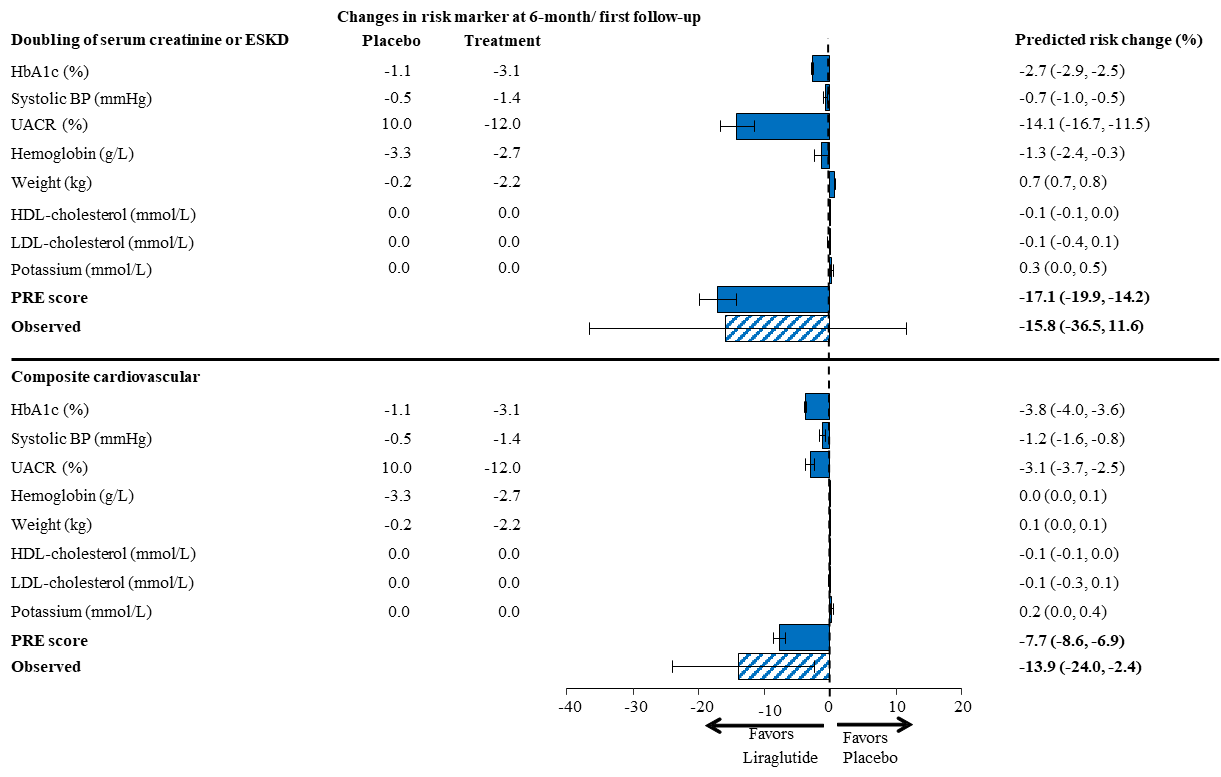


**Supplementary Figure 4.** Predicted risk change for the composite kidney (top) and the composite cardiovascular (bottom) outcomes in the total population with all risk markers measured at baseline and 24-month (N=9340). Bars indicate estimates of the mean change in relative risk for specific outcomes with 95% confidence interval, as compared to placebo. HbA1c, glycated hemoglobin; UACR, urinary-albumin-creatinine-ratio; BP, blood pressure; HDL, high-density-lipoprotein; LDL, low-density-lipoprotein; PRE score, Parameter Response Efficacy score.


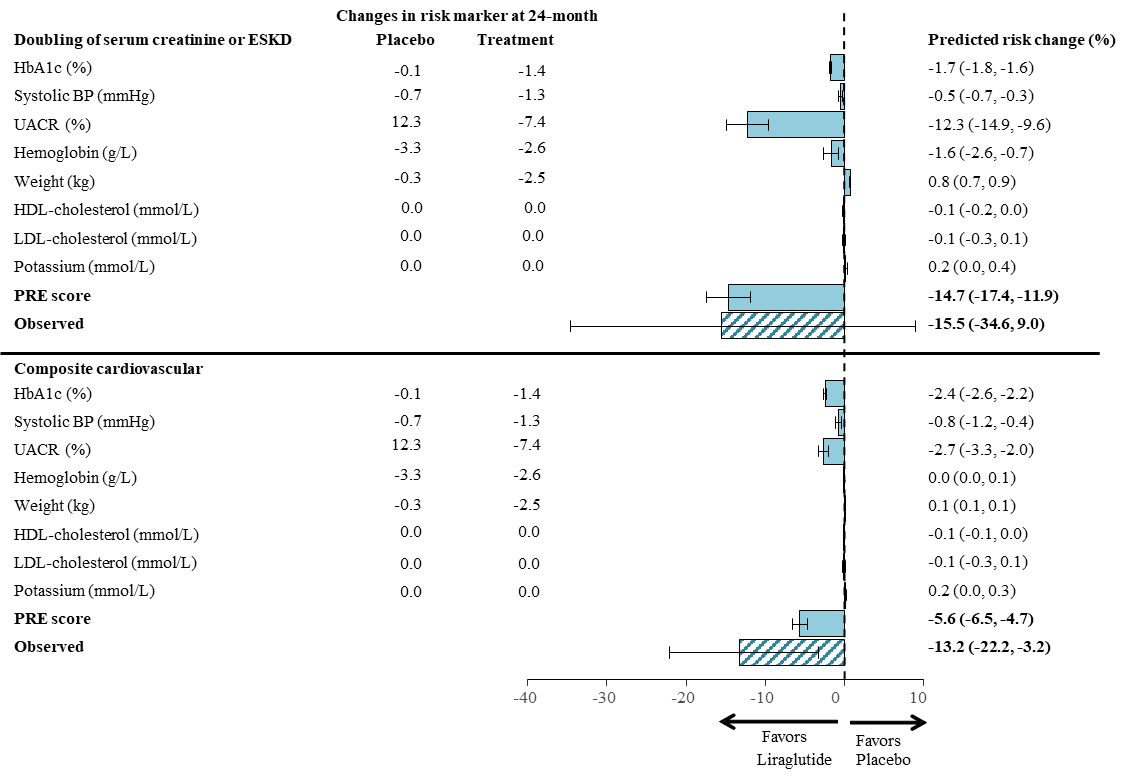

Supplement: Supplementary file 1 [file DataSheet1.docx]
